# Spatial analysis of MODIS aerosol optical depth, PM_2.5_, and chronic coronary heart disease

**DOI:** 10.1186/1476-072X-8-27

**Published:** 2009-05-12

**Authors:** Zhiyong Hu

**Affiliations:** 1 Department of Environmental Studies, University of West Florida, Pensacola, FL, USA

## Abstract

**Background:**

Numerous studies have found adverse health effects of acute and chronic exposure to fine particulate matter (PM_2.5_). Air pollution epidemiological studies relying on ground measurements provided by monitoring networks are often limited by sparse and unbalanced spatial distribution of the monitors. Studies have found correlations between satellite aerosol optical depth (AOD) and PM_2.5 _in some land regions. Satellite aerosol data may be used to extend the spatial coverage of PM_2.5 _exposure assessment. This study was to investigate correlation between PM_2.5 _and AOD in the conterminous USA, to derive a spatially complete PM_2.5 _surface by merging satellite AOD data and ground measurements based on the potential correlation, and to examine if there is an association of coronary heart disease with PM_2.5_.

**Results:**

Years 2003 and 2004 daily MODIS (Moderate Resolution Imaging Spectrometer) Level 2 AOD images were collated with US EPA PM_2.5 _data covering the conterminous USA. Pearson's correlation analysis and geographically weighted regression (GWR) found that the relationship between PM_2.5 _and AOD is not spatially consistent across the conterminous states. The average correlation is 0.67 in the east and 0.22 in the west. GWR predicts well in the east and poorly in the west. The GWR model was used to derive a PM_2.5 _grid surface using the mean AOD raster calculated using the daily AOD data (RMSE = 1.67 *μg/m*^3^). Fitting of a Bayesian hierarchical model linking PM_2.5 _with age-race standardized mortality rates (SMRs) of chronic coronary heart disease found that areas with higher values of PM_2.5 _also show high rates of CCHD mortality:  = 0.802, posterior 95% Bayesian credible interval (CI) = (0.386, 1.225).

**Conclusion:**

There is a spatial variation of the relationship between PM_2.5 _and AOD in the conterminous USA. In the eastern USA where AOD correlates well with PM_2.5_, AOD can be merged with ground PM_2.5 _data to derive a PM_2.5 _surface for epidemiological study. The study found that chronic coronary heart disease mortality rate increases with exposure to PM_2.5_.

## Background

Numerous epidemiological studies have indicated that exposure to fine particulate matter (particles smaller than 2.5 micrometers, PM_2.5_) is associated with asthma, respiratory infections, lung cancer, cardiovascular problems, and premature death [1-5]. A few have examined coronary heart disease, finding evidence for acute effects on mortality and hospital admissions [6-8]. Recently, attention has focused on whether there is an association between chronic exposure to air pollution and coronary heart disease [9]. An ecological study at the census enumeration district level found an association between nitrogen oxides, and to a lesser extent particulate matter (PM_10_) and carbon monoxide, and coronary heart disease mortality in Sheffield, UK [9]. The objective of this study was to examine if there is an association of coronary heart disease with chronic exposure to PM_2.5_. The study adopted an ecological method using aggregate disease data at the county level and average PM_2.5 _concentration value for each county.

Air pollution epidemiological studies often rely on ambient observations to provide metrics of exposure, as in studies of PM and cardiovascular diseases [2,4,9-12]. Methods of exposure assessment in those studies include averaging multiple monitors within each enumeration unit or study site [4,10,11], assigning the exposure value of the nearest monitor to each case/control [2,12] and spatial interpolation/modelling method [9]. Ground monitoring data often lacks spatially complete coverage. Ground monitors are rare in rural areas. Assessment of the exposure to air pollution using in situ observations is hampered by the sparse and unbalanced spatial distribution of the monitors.

The repetitive and broad-area coverage of satellites may allow atmospheric remote sensing to offer a unique opportunity to monitor air quality at continental, national and regional scales. Recent studies have established quantitative relationships between satellite derived aerosol optical depth (AOD), which describes the mass of aerosols in an atmospheric column, and PM_2.5 _using linear regression models [13-23]. Except long-range dust or pollution transport events, AOD is dominated by near-surface emissions sources [24]. AOD retrieved at visible wavelengths is most sensitive to particles between 0.1 and 2 micrometers [25]. Several studies have merged AOD with ground PM_2.5 _measurement to derive PM_2.5 _surfaces [26-28]. A study in a region centered in Massachusetts [26] examined the benefits of using AOD retrieved by the Geostationary Operational Environmental Satellite (GOES) in conjunction with land use and meteorological information to estimate ground-level PM_2.5 _concentrations. Another project [27] combined MODIS (Moderate Resolution Imaging Spectrometer) AOD data with US EPA PM_2.5 _data to estimate a PM_2.5 _surface in Atlanta, Georgia. Existing studies estimating PM_2.5 _surfaces using AOD data use uniform linear relationships between AOD and PM_2.5_. However, studies have found that correlation between PM_2.5 _and AOD is not spatially consistent [29] due to variation in terrain, land cover, selection of aerosol model in the AOD retrieval algorithm and meteorological factors.

This paper quantitatively examined the relationship between PM_2.5 _ground measurements and MODIS AOD data in the conterminous USA using Pearson's correlation analysis and geographically weighted regression (GWR). For the region with high correlations, the GWR model was used to calculate a PM_2.5 _surface based on the AOD data and the spatially varying relationships between PM_2.5 _and AOD. A Bayesian hierarchical simulation model was used to link chronic coronary heart disease mortality rate with PM_2.5_.

## Methods

### MODIS data

The MODIS sensor flies on polar-orbiting and sun-synchronous Terra and Aqua satellites. MODIS performs measurements in the visible to thermal infrared spectrum region. The MODIS sensor was expected to be the key for monitoring global aerosol properties. Not only have MODIS aerosol products been used to answer scientific questions about radiation and climate, they are being used for applications not previously intended, including monitoring surface air quality for health [15,16,30-32].

One of the fundamental aerosol products from MODIS is spectral AOD. The MODIS level 2 files are produced everyday at the spatial resolution of a 10 km × 10 km (at nadir) and represent the first level of MODIS aerosol retrieval. The latest version of the MODIS aerosol retrieval algorithm is Collection 5 (C005) [33]. The aerosol retrieval makes use of seven wavelength bands (channels 1 to 7), and a number of other bands to help with cloud rejection and other screening procedures. The aerosol algorithm relies on calibrated, geolocated reflectance Level 1B data. In addition, the MODIS algorithm uses cloud mask product [34], atmospheric profile product and ancillary data from NCEP (National Center for Environmental Prediction) analyses, including hourly meteorological analysis and daily ozone analysis. Different dynamic aerosol models (biomass burning, dust aerosol, and aerosol from industrial/urban origin) are used to determine the aerosol optical properties used in the algorithm for different parts of the land. Over the land of the conterminous US, the aerosol retrieval algorithm uses the urban/industrial aerosol model for the east and the biomass burning/dust model for the west. The splitting line is at approximately -100° longitude.

Daily level 2 MODIS data (2003–2004) were obtained from the NASA Level 1 and Atmosphere Archive and Distribution System (LAADS Web) [35]. A two-year average AOD raster data layer (10 km by 10 km grid) was calculated. Data from both Terra and Aqua satellites were used. MODIS AOD data are not available every day due to cloud cover. Data for cold seasons (October to March) were not used in the two-year average calculation and correlation analysis. Cloud cover, snow reflectivity, and diminished vertical mixing all reduce the accuracy of ground-level pollutant levels measured in winter. During warm seasons, vertical columns in the atmosphere are more integrated. AOD measures correlate best with ground-based monitoring in warm months, likely because of stronger boundary layer mixing during the warmer months [36].

### Pearson's correlation and geographically weighted regression

PM_2.5 _ground data was obtained from US EPA Air Quality System (AQS) online Data Mart [37]. There were 877 monitoring sites. PM_2.5 _data was collated with AOD both temporally and spatially. For each MODIS AOD image scene and each monitor within the scene, PM_2.5 _measurement within one hour of the imaging time was assigned to the pixel containing the monitor. Pearson's correlation value was calculated for each monitoring site. The Pearson's correlation analysis examined the temporal relationship between PM_2.5 _and AOD for each site. The relationship shows how AOD changes as PM_2.5 _changes over time at a sampling site. To visualize the spatial variation of the correlations, inverse distance weighted interpolation was used to create a correlation surface. It should be noted that the correlation surface was used for visualization purpose only. It was not intended for the use of quantitative analysis, since the interpolation was based on the unbalanced distribution of the monitors and the accuracy was not spatially consistent.

A geographically weighted regression (GWR) model was also fitted to examine the relationship between PM_2.5 _(dependent variable) and AOD (independent variable) using the two-year average PM_2.5 _and AOD values. The mean PM_2.5 _value was geo- collated with the mean AOD value for each of the 877 sites. GWR is a local form of linear regression used to model spatially varying relationships [38]. GWR is one of several spatial regression techniques, increasingly used in geography and other disciplines. GWR provides a local model of the variable or process we are trying to understand/predict by fitting a regression equation to every feature in the dataset. GWR constructs these separate equations by incorporating the dependent and explanatory variables of features falling within a kernel of each target feature. GWR accounts for the effect of local multicolliearity which occurs when the values for a particular explanatory variable cluster spatially. GWR creates coefficient raster surfaces for the model intercept and each explanatory variable. GWR used in the study analyzed the spatial varying relationship between two-year mean AOD and mean PM_2.5_. Like the correlation surface created from Pearson's correlation analysis, GWR revealed how the relationship between AOD and PM_2.5 _changes across the space. Pearson's correlation analysis used daily time-series of data, and GWR used mean data. While both analyses revealed the spatial variation of the relationship between AOD and PM_2.5_, GWR also calculated a mean PM_2.5 _surface that was needed for disease modeling since disease data was a two-year aggregate. In this study, a fixed kernel was used to fit the regression. The bandwidth value was chosen by using the corrected Akaike Information Criterion (AICc). This option tries to identify an optimal fixed distance. For the region with high correlations, the coefficient raster surfaces were applied to the two-year mean AOD raster to calculate a PM_2.5 _surface. The accuracy of the estimated PM_2.5 _surface was assessed by comparison to the two-year mean PM_2.5 _ground measurements.

### Disease data

Modelling of disease and PM_2.5 _was focused on the region where higher positive correlation between AOD and PM_2.5 _was found. Chronic coronary heart disease (CCHD) (International Classification of Disease version 10 codes: I25.0-I25.6, I25.8, I25.9) data at the county level were extracted for the period from 2003 to 2004 from the National Center for Health Statistics Compressed Mortality File 1999–2005 in the CDC WONDER online database [39]. CCHD mortality count and population at risk was retrieved by county, race (White, Black or African American, Other race) and age (5 years interval from 1–4 to 85+). Aggregated CCHD mortality count and population at risk were also retrieved by race and age groups for the focused study area to be used as standard population in calculating CCHD mortality rate adjusting for race and age effects.

Race and age adjusted rate was calculated using indirect standardization [40] for each county. Rate adjustment is a technique for removing the effects of race and age from crude rates, so as to allow meaningful comparisons across populations with different underlying race and age structures. In the US, researchers normally use the year 2000 US decennial census data as a standard population to standardize rates. In the present study, the use of an internal standard population consisting of race-age-specific rates from a super-population containing the regions to compare would be better than an external standard based on individuals from an entirely separate population [41]. The indirect standardization first calculated expected number of CCHD deaths for each county. The calculated death count is the number of cases that would be expected in the study population if people in the study population contracted the disease at the same rate as people in the standard population. Standardized mortality rates (SMRs) were calculated by dividing the observed count by the expected value. According to Curtin and Klein [42], one of the problems with rate adjustment is that rates based on small numbers of deaths will exhibit a large amount of random variation. In the aggregate CCHD mortality count and population data set, counts of twenty or less were flagged as statistically "unreliable". CCHD counts were "suppressed" when the data meets the criteria for confidentiality constraints. Counts for counties with census year populations of less than 100,000 were replaced with "suppressed" if the number of cases is five or less and the count is based on only one or two years of data. All unreliable and suppressed data were not used in calculating standardized rates and spatial analysis and modelling thereafter.

### Bayesian hierarchical modelling of CCHD and PM_2.5_

A GIS zonal statistical function was used to calculate the mean PM_2.5 _value for each county by averaging PM_2.5 _values of all cells within the county. A Bayesian hierarchical model was used to explore the association between CCHD mortality and PM_2.5_. Simulation-based algorithms for Bayesian inference allow us to fit very complicated hierarchical models, including those with spatially correlated random effects. In this ecological study, there could exist spatial autocorrelation within values of the CCHD mortality and PM_2.5_. The following model was fitted allowing a convolution prior for the random effects:



where *i *is the index for a county, *O *is observed CCHD death count, *E *is expected death count reflecting race-age-standardized values. For model specification, an improper (flat) prior for the intercept parameters β_0 _and a uniform prior distribution for the fixed-effect (β_1_) were assumed. By fixed effect we mean it applies equally to all the counties. Two sets of county-specific random effects were included in the model. The first set *b*_*i *_is spatially structured random effects assigned an intrinsic Gaussian conditional auto-regression (CAR) prior distribution [43]. The second set of random effects *h*_*i *_is assigned an exchangeable (non-spatial) normal prior. The random effect for each county is thus the sum of a spatially structured component *b*_*i *_and an unstructured component *h*_*i*_. This is termed a convolution prior [43,44]. The model is more flexible than assuming only CAR random effects, since it allows the data to decide how much of the residual disease risk is due to spatially structured variation, and how much is unstructured over-dispersion. To complete the model specification, conjugate inverse-gamma prior distributions were assigned to the variance parameters associated with the exchangeable and/or CAR priors.

The Markov chain Monte Carlo (MCMC) simulation computation technique and Gibbs sampling algorithm were used to fit the Bayesian model. Having specified the model as a full joint distribution on all quantities, whether parameters or observables, we wish to sample values of the unknown parameters from their conditional (posterior) distribution given those stochastic nodes that have been observed. MCMC methods perform Monte Carlo simulations generating parameter values from Markov chains having stationary distributions identical to the joint posterior distribution of interest. After these Markov chains converge to a stationary distribution, the simulated parameter values represent a correlated sample of observations from the posterior distribution. The basic idea behind the Gibbs sampling algorithm is to successively sample from the conditional distribution of each node given all the others. Under broad conditions this process eventually provides samples from the joint posterior distribution of the unknown quantities. Summaries of the post-convergence MCMC samples provide posterior inference for model parameters. The result of such analysis is the posterior distribution of an intensity function with covariate effects.

The model was fitted using the *WinBUGS *software – an interactive Windows version of the *BUGS *(Bayesian inference Using Gibbs Sampling) program for Bayesian analysis of complex statistical models using MCMC techniques [45]. A queen's case spatial adjacency matrix (*w*_*ij *_= 1 when county *i *and *j *share a boundary or a vertice, *w*_*ij *_= 0 otherwise) that is required as input for the conditional autoregressive distribution was created using the Adjacency for WinBUGS Tool developed by Upper Midwest Environmental Sciences Center of the US Geological Survey (USGS) [46]. Typical disease mapping applications consider adjacency-based weights. Other weighting options (e.g., distance-decay weights) also appear in the literature (e.g., [47]) but are much less widely applied. Two Markov chains were simulated in the present study. The MCMC samplers were given initial values for each stochastic node. A total of 80,000 iterations with 60,000 burn-in were run. "Burn-in" denotes iterations that were discarded due to non-convergence of the Markov chains at the early stages of the simulation. A visual inspection of the Markov chain trace plots of the sample values versus iteration has indicated that convergence has been reached after 60,000 iterations. Inference was based on iterations 60,001 to 80,000.

## Results

### Pearson's correlation

**Figure 1:**
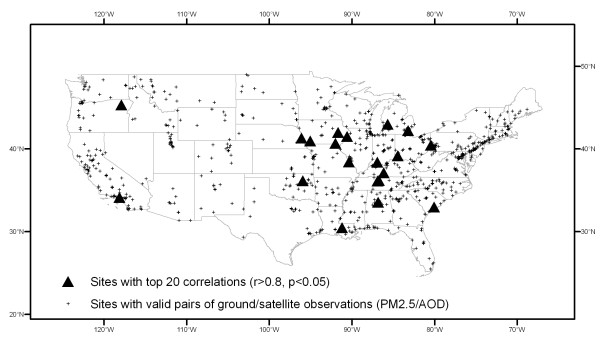
**Monitoring sites with top 20 correlations of PM_2.5 _with AOD**.

**Figure 2:**
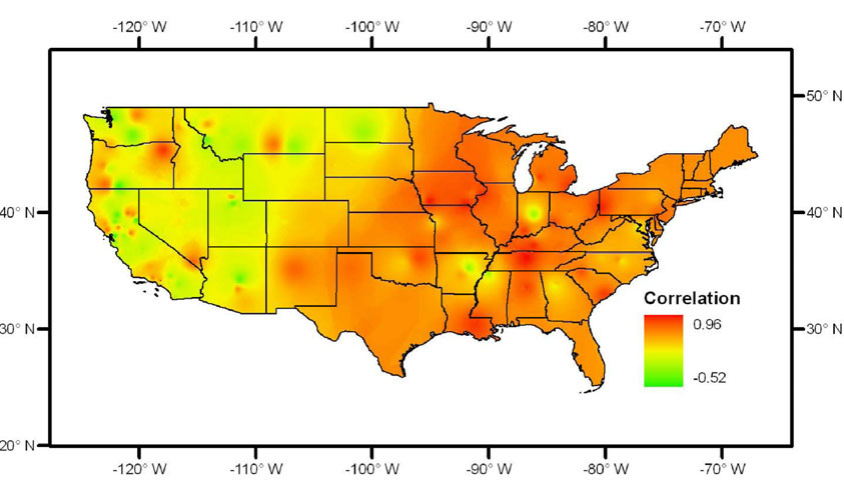
**Surface of Pearson's correlation between PM_2.5 _and AOD**.

Figure 1 shows the 20 monitoring sites with top significant correlations (r > 0.8, p < 0.05). Eighteen of the twenty sites are located east of the -100° longitude line. The average correlation is 0.67 in the east and 0.22 in the west. The highest correlation (r = 0.96) is at Nashville, Tennessee. The interpolated correlation surface is shown in Figure 2. The surface indicates that the eastern part of the USA has higher correlation between PM_2.5 _and AOD.

### GWR

**Figure 3:**
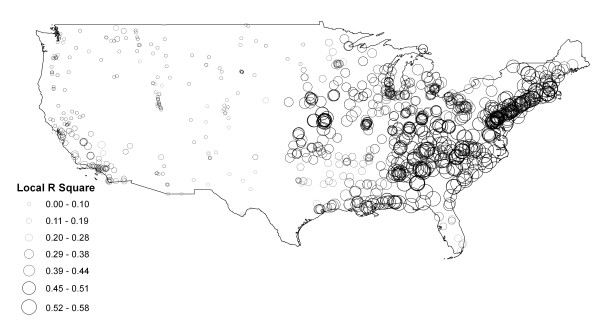
**Local R square of geographically weighted regression**.

**Figure 4:**
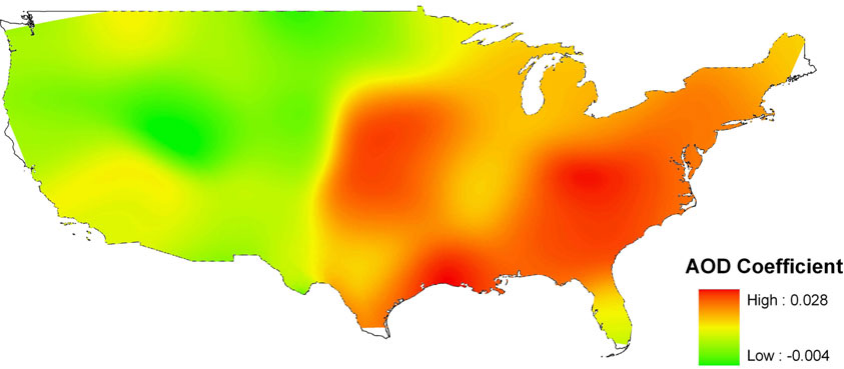
**Coefficient raster surface for AOD from geographically weighted regression**.

Results from the GWR show that all the monitoring sites have a condition number less than 30. The condition number evaluates local collinearity. In the presence of strong local collinearity, results become unstable. Results associated with condition numbers larger than 30 may be unreliable. Figure 3 shows a map of local R square. R^2 ^values range between 0.0 and 1.0 and indicate how well the local regression model fits observed PM_2.5 _values. Very low values indicate the local model is performing poorly. It can be seen that GWR predicts well in the eastern USA and poorly in the west. Figure 4 shows the coefficient raster surface for the explanatory variable AOD. The map exhibits regional variation in the explanatory variable. The relationship between PM_2.5 _and AOD is not spatially consistent (stationary) across the conterminous states. Like Pearson's correlation surface, eastern USA shows higher AOD coefficient values, while values in the west are lower. Even negative values are found in part of the western region. The Pearson's correlation and GWR analyses revealed that it is appropriate to estimate a PM_2.5 _surface using AOD for disease modelling for the region to the east of the -100° longitude line. Application of the GWR model to the two-year mean AOD raster resulted in a continuous PM_2.5 _surface with a root mean square error (RMSE) of 1.67 *μg/m*^3^. Compared to the average PM_2.5 _value of 10.18 *μg/m*^3 ^for all the monitors, the PM_2.5 _estimation achieved an accuracy of 84%.

### Bayesian model

**Figure 5:**
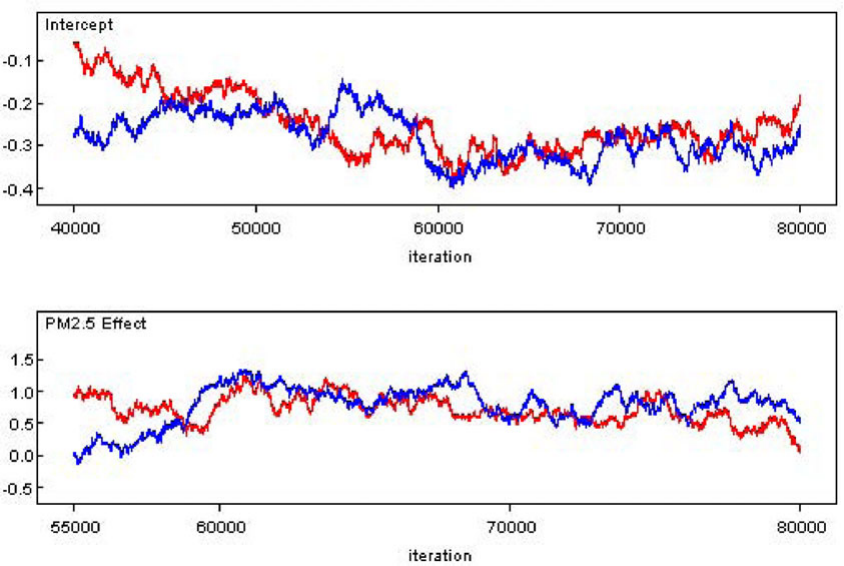
**Trace plots of the 80,000 Markov Chain Monte Carlo (MCMC) updates**. Simulation trace plots for the intercept and the effect of PM_2.5 _on chronic coronary heart disease from the Bayesian hierarchical model with a convolution prior. Horizontal axis represents iteration number and vertical axis represents simulated parameter value. The red trace is for one Markov chain, and the blue for the other.

**Figure 6:**
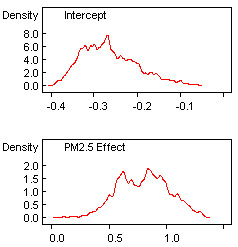
**Kernel estimates of the posterior densities of the fixed effects in the Bayesian hierarchical model**. Horizontal axis represents simulated parameter values and vertical axis represents the density of each value.

The number of data points (counties) in the Bayesian modelling is 2,306. The actual number of counties to the east of the -100 degree longitude is 2,506. Some counties with unreliable and suppressed disease data were omitted from the analysis. Figure 5 displays a trace of the 80,000 updates of the fixed effects in each of model parameters in β = (β_0_, β_1_)'. The horizontal axis represents the simulation iteration number. The vertical axis represents simulated parameter values. Positive parameter β_1 _values indicate positive relationship between PM_2.5 _and CCHD mortality. The red trace is for one Markov chain, and the blue for the other. For each of the two parameters (the intercept and the PM_2.5 _effect), the simulation moves away from the initial seed values. After 60,000 updates, each simulation generates values fluctuating around within a consistent range of values representing the posterior distribution of each model parameter, indicating that convergence has been reached. Inferences were made about the parameters of the model using the simulated values on iterations 60,001 to 80,000. Table 1 provides the estimated posterior mean, median, and associated 95% credible set for each of the fixed effects. A 95% credible set defines an interval having a 0.95 posterior probability of containing the parameter of interest which is assumed to be a random variable in Bayesian statistics. The 2.5%, 50% (median) and 97.5% quantiles and posterior mean were calculated via an approximate algorithm [48]. Summaries of the postconvergence MCMC samples provide posterior inference for model parameters. For example, the sample mean of the postconvergence sampled values for a particular model parameter provides an estimate of the marginal posterior mean and a point estimate of the parameter itself. The interval defined by the 2.5^th ^and 97.5^th ^quantiles of the postconvergence sampled values for a model parameter provides a 95% interval estimate of the parameter. Standard deviations and Monte Carlo (MC) errors were calculated to assess the accuracy of the simulation. The MC error is an estimate of the difference between the mean of the sampled values and the true posterior mean. As a rule of thumb, the simulation should be run until the Monte Carlo error for each parameter of interest is less than about 5% of the sample standard deviation. The MC errors calculated from the iterations 60,001 to 80,000 for both the parameters are less than 5% of the corresponding standard deviations, suggesting an accurate posterior estimate for each parameter. In addition, Figure 6 provides kernel estimates of the corresponding posterior densities. The horizontal axis represents simulated parameter value. The vertical axis represents the density of each parameter value. The results in Figures 5 and 6 and Table show an association between PM_2.5 _pollution on CCHD. The 95% credible set covers positive values:  = 0.802, CI = (0.386, 1.225). The positive boundary values of the CI indicate that areas with higher values of PM_2.5 _also show high rates of CCHD mortality.

**Table 1:** Results of Bayesian hierarchical modeling

Fixed effects	Posterior mean	Posterior median	Standard deviation	MC error	95% Credible set
β_0_	-0.264	-0.273	0.064	0.003	(-0.366, -0.117)
β_1_	0.802	0.812	0.223	0.010	(0.386, 1.225)

* Posterior means, medians, and 95% credible sets are based on 20,000 post-convergence iterations (from 60,0001 to 80,000). Fixed effects are: β_0 _– intercept, β_1 _– effect of PM_2.5_.

## Discussion

The Pearson's correlation and GWR analyses found that the relationship and between PM_2.5 _and MODIS AOD is not spatially consistent across the conterminous states. In the west AOD poorly correlates with PM_2.5_. The east region exhibits high positive correlations between PM_2.5 _and AOD where a PM_2.5 _surface can be estimated using AOD data for assessment of PM_2.5 _effect on disease. The correlation variation is likely due to the difference in terrain, AOD retrieval algorithm [29] and meteorological conditions. The algorithm to calculate AOD is based on dark surface pixels, contrast with surface, and assumptions in the model on the types of pollutants and the terrain [33]. The lower correlations in general can be found in the more arid parts as in the west of the USA where the higher surface reflectance reduces contrasts and where the model assumes more dust and smoke than urban/industrial model. The MODIS aerosol retrieval algorithm uses the urban/industrial aerosol model for eastern USA and other densely populated regions, such as Western Europe and eastern China [49]. Urban-industrial aerosols are mainly from fossil fuel combustion in populated industrial regions and dominated by fine particles. The positive relationship in the east could exist in other regions of the world where the aerosol retrieval algorithm uses the urban/industrial aerosol model. AOD measurements calculated from the sulphate-rich urban/industrial aerosol model may be more useful in predicting ground-based particulate matter levels. The inherent differences in the datasets may also explain some of the correlation variation [29]. MODIS AOD measures aerosol scattering in a total column from ground to satellite while EPA PM monitors measure a size-based subset of PM in the boundary layer in a specific location.

The Bayesian model found a spatial association between age-race-standardized mortality rate of chronic coronary heart disease and PM_2.5_. There is an excess risk of CCHD mortality in areas with high PM_2.5 _levels. Fine aerosol particles tend to penetrate into the gas-exchange regions of the lung, and ultrafine particles are able to pass through the lungs to enter the blood circulation and affect other organs [50]. A study indicates that PM_2.5 _leads to high plaque deposits in arteries, causing vascular inflammation and atherosclerosis – a hardening of the arteries that reduces elasticity, which can lead to heart attacks and other cardiovascular problems [51,52].

This study has several strengths. First, the present study calculated PM_2.5 _surface by merging satellite derived AOD with ground measurements using GWR. To our knowledge, no applications of satellite remote sensing data in spatial modeling of regional PM_2.5 _concentrations have considered spatially varying relationships. Existing studies often use a uniform linear relationship to estimate PM_2.5 _using AOD. While the uniform relationship could apply to a smaller region (such as a metropolitan area), it might not work for a larger region (such as the eastern USA including 37 states in the study). Second, ecological studies are particularly useful when disease data at individual level is not available and individual level of exposure is either difficult or impossible to obtain, or can be only measured imprecisely [47]. Ecological studies are more useful for generating and testing hypothesis [53]. The association between CCHD mortality rate and PM_2.5 _revealed by the Bayesian model can be taken as indicative (though not necessarily causative) of a potential air pollution effect. Third, the MCMC computation techniques and Bayesian analysis allow us to use more elaborate models without conventional model restrictions. The model also allows us to treat the CCHD death counts as outcomes without the awkward transformations of the outcome and covariates of interest required to meet standard assumptions for linear regression models. Fourth, the MODIS aerosol data lends itself to population-based exposure assessment and the ecological approach. Moderate resolution satellite image pixels share the same feature dimension with the disease data enumeration districts in that both pixels and county polygons are area features. Derivation of a continuous PM_2.5 _surface using AOD alleviates the non- straightforwardness and noncompliance of comparing discrete point monitoring data with area-aggregated disease data. A continuous PM_2.5 _surface calculated by merging AOD and ground data accounting for varying correlations better represents aggregate exposure than averaging ground measurements over a limited number of monitoring sites.

There are also several limitations to this study. First, the correlation and GWR analyses did not account for meteorological parameters (such as mixing height, relative humidity, air temperature, and wind speed), aerosol vertical distribution, and aerosol properties. Although AOD is a highly significant predictor of PM_2.5_, those parameters could also modify the relationship between AOD and PM_2.5 _concentrations. Second, the study estimated an ambient PM_2.5 _surface to assess the health effect of fine particulate air pollution. However, ambient concentrations do not necessarily represent actual exposures, which can be influenced by the infiltration of ambient concentrations into indoor facilities (such as automobiles, homes, schools, and work places) and the activity of individuals (such as outdoor exercise, walking, commuting, etc.). Third, the use of aggregated data and therefore inferences based on the analysis cannot be directly transferred to the individual level. An inherent limitation of an ecological study is that it uses aggregate data and does not have the ability to incorporate individual information. The population-based ecological analysis did not consider population dynamics, for example, population migration. Fourth, chronic heart disease is a long term medical condition that has been shown to progress at varying rates over a lifetime with many other environmental, biomedical and social factors in effect. The study examined chronic heart disease over a short timeframe. Last, it must be noted that there are limitations of AOD data. AOD is a quantitative measure of total column aerosol, which is the mass of aerosols within a measured column extending from Earth to the satellite sensor. AOD does not correlate well with PM_2.5 _during cold seasons. This study used warm season AOD data but the disease data covered whole seasons. AOD does not differentiate between fine-mode and course-mode particles. Ideally, fine-mode optical depth should be used for the health effect assessment because fine mode particles which dominate urban/industrial pollution cause the most severe health problem. However, land-based measures of fine-mode fraction of AOD can only be used as a qualitative indicator of whether AOD values are dominated by natural or anthropogenic emissions [33]. Additionally, AOD does not specify the location of aerosols within a column and the AOD values do not necessarily represent ground conditions. This lack of vertical information emphasizes the importance of combining the satellite image with vertical profiles. Emerging Light Detection and Ranging (LIDAR) systems could provide vertical resolution for AOD and quantify pollutant levels on the ground.

## Conclusion

There is a spatial variation of the relationship between PM_2.5 _and AOD in the conterminous USA. In the eastern USA where AOD correlates well with PM_2.5_, AOD can be merged with ground PM_2.5 _data to derive a PM_2.5 _surface for epidemiological study. The study found that chronic coronary heart disease mortality rate increases with exposure to PM_2.5_. High risk of CCHD mortality was found in areas with elevated levels of fine particulate air pollution. The association between CCHD mortality and PM_2.5 _would justify the need of further toxicological approach for investigating the biological mechanism of the adverse effect of fine particulate air pollution on heart. The evidence of raised CCHD mortality risk in high pollution areas would support targeting of policy interventions on such areas to reduce pollution levels. Aerosol remote sensing could help fill pervasive data gaps that impede efforts to study air pollution and protect public health.

## Competing interests

The author declares that they have no competing interests.

## Authors' contributions

ZH conceived of the study, conducted the research and wrote the manuscript.
